# Discovering patterns in drug-protein interactions based on their fingerprints

**DOI:** 10.1186/1471-2105-13-S9-S4

**Published:** 2012-06-11

**Authors:** Weimin Luo, Keith CC Chan

**Affiliations:** 1 Department of Computing, The Hong Kong Polytechnic University, Hong Kong, China

## Abstract

**Background:**

The discovering of interesting patterns in drug-protein interaction data at molecular level can reveal hidden relationship among drugs and proteins and can therefore be of paramount importance for such application as drug design. To discover such patterns, we propose here a computational approach to analyze the molecular data of drugs and proteins that are known to have interactions with each other. Specifically, we propose to use a data mining technique called *Drug-Protein Interaction Analysis *(*D-PIA*) to determine if there are any commonalities in the fingerprints of the substructures of interacting drug and protein molecules and if so, whether or not any patterns can be generalized from them.

**Method:**

Given a database of drug-protein interactions, *D-PIA *performs its tasks in several steps. First, for each drug in the database, the fingerprints of its molecular substructures are first obtained. Second, for each protein in the database, the fingerprints of its protein domains are obtained. Third, based on known interactions between drugs and proteins, an interdependency measure between the fingerprint of each drug substructure and protein domain is then computed. Fourth, based on the interdependency measure, drug substructures and protein domains that are significantly interdependent are identified. Fifth, the existence of interaction relationship between a previously unknown drug-protein pairs is then predicted based on their constituent substructures that are significantly interdependent.

**Results:**

To evaluate the effectiveness of *D-PIA*, we have tested it with real drug-protein interaction data. *D-PIA *has been tested with real drug-protein interaction data including enzymes, ion channels, and protein-coupled receptors. Experimental results show that there are indeed patterns that one can discover in the interdependency relationship between drug substructures and protein domains of interacting drugs and proteins. Based on these relationships, a testing set of drug-protein data are used to see if *D-PIA *can correctly predict the existence of interaction between drug-protein pairs. The results show that the prediction accuracy can be very high. An AUC score of a ROC plot could reach as high as 75% which shows the effectiveness of this classifier.

**Conclusions:**

*D-PIA *has the advantage that it is able to perform its tasks effectively based on the fingerprints of drug and protein molecules without requiring any 3D information about their structures and *D-PIA *is therefore very fast to compute. *D-PIA *has been tested with real drug-protein interaction data and experimental results show that it can be very useful for predicting previously unknown drug-protein as well as protein-ligand interactions. It can also be used to tackle problems such as ligand specificity which is related directly and indirectly to drug design and discovery.

## Background

In many different and extremely complex ways, the chemical pathways in our bodies are affected by various diseases. When one is sick, it might be a mistake in one reaction in a pathway that stops an important protein from being produced or causes too much of it to be produced. To correct such mistakes, drug molecules can be developed to interact with *target *protein molecules to activate or inhibit some of its functions thereby causing a protein to be produced more, or less. To facilitate drug design and discovery, it would therefore be very useful if we can predict whether or not a particular drug candidate may interact with a particular target protein based on its their structures at the molecular or sub-molecular levels.

Over the past decade, a lot of effort has been made to investigate into how drug and protein interact and the most notable among the work done are those related to protein-ligand docking [1]. Ligand is a molecule that binds to another chemical entity to form a large complex and protein-ligand docking is concerned with the prediction of the position and orientation of a ligand for binding with a protein receptor. If a ligand candidate that binds with a certain target can be found, drug molecules can then be designed to contain this ligand. However, the finding of such ligand candidate is difficult as protein-ligand docking requires knowledge about the 3D structures of the proteins and obtaining such knowledge can be very difficult [2].

Instead of investigating into protein-ligand docking, there has also been some effort to look into the analysis of molecular substructures [3] and biological activities [4]. In [3], for example, the concept of "privileged" substructures is introduced as chemical substructures that are commonly present in many drugs. In other words, in predicting if a drug may have any interaction with a protein, one can search for the presence of such privileged substructures in the drug molecules as an indicator of the likelihood of the existence of an interaction relationship with a protein. While such approach to finding privileged substructures may sound reasonable, it is considered controversial as abundance of drug structures may be a trivial consequence of their abundance in biochemical molecules.

Other than finding privileged substructures, a variety of statistical methods have recently been proposed to predict drug-target or more generally, protein-ligand interactions [5,6]. There have also been some attempts to mine structural patterns from biological or biochemical data based on molecular fingerprints. The concept of molecular fingerprints, which is first introduced in [7], refers to the representation of chemical structures originally designed to assist in chemical database search. They become so widely used later on for data analysis tasks such as similarity search [8], clustering [9], and classification [10]. Molecular fingerprints have been used in such tasks to encode a wide range of 2D and 3D structural or conformational features of the molecules. A novel method for representing and analyzing 3D protein-ligand binding interactions, for example, is proposed in [11]. The key to the proposed method is to analyse the fingerprints obtained from translating the 3D structural binding information from a protein-ligand complex into a one-dimensional binary string.

Most of the work mentioned above has been performed independently from the viewpoints of either ligands or proteins. Not much work has been done to investigate into how the chemical and biological space may interact with each other. In [2], the paper reports on some attempts made to try to connect the two space. It proposes an approach to extract drug substructures and protein domains from a drug-protein interactions dataset by encoding chemical substructures of the drugs and the proteins domains of the dataset into molecular fingerprints. The paper explains how sparse canonical correspondence analysis (SCCA) can be performed on the data. As pointed out in the paper, the effectiveness of the proposed approach depends very much on the correct setting of a number of predefined parameters and the method may not work well when sparsity of data is not a relevant characteristic.

To identify ligand candidates efficiently for such applications as drug design and discovery, we need to be able to predict if a drug may interact with a protein without having to obtain full information of the 3D structures of protein molecules at an early stage. To do so, we propose to use a data mining algorithm called *D-PIA *(Drug-Protein Interaction Analysis). Instead of relying on the availability of the 3D structural information of a target protein to predict if it may have any interaction with a certain drug candidate, *D-PIA *only makes use of the 2D molecular fingerprints of the protein in the prediction process.

Proteins are molecules consisting of a long chain of amino acids with unique structures and substructures. A protein domain is a part of a protein chain that can evolve, function, and exist independently of the rest of the other parts of the chain [12]. *D-PIA *performs its tasks by first breaking down drug molecules into substructures and proteins into their protein domains. By so doing, *D-PIA *attempts to determine if the drug substructures may interact or bound with the protein domains and if the strength of such interactions or bindings may determine if drugs can be designed for optimal compatibility with the human body and with other drugs [13].

Once the drug substructures and protein domains are identified, *D-PIA *makes use of a probabilistic measure to determine if a drug substructure and a protein domain are interdependent on each other and it does so in several steps: (i) for each drug in the database, the fingerprints of its molecular substructures are first obtained; (ii) for each protein in the database, the fingerprints of its protein domains are obtained; (iii) based on known interactions between drugs and proteins, an interdependency measure between the fingerprint of each drug substructure and protein domain is then computed; (iv) based on the interdependency measure, drug substructures and protein domains that are significantly interdependent are identified; and (v) the existence of interaction relationship between a previously unknown drug-protein pairs is then predicted based on their constituent substructures that are significantly interdependent.

*D-PIA *has been tested with real data involving two thousand drugs and the proteins that they interact with. Our experimental results show that it can be very helpful for predicting drug-protein and protein-ligand interactions. It can also be used to address problems such as ligand specificity.

## Methods

Suppose that we have a set of *M *drugs {*D_1_, D_2_, ... D_i_, .. D_M_*} with each characterized by *p *substructure descriptors respectively. Suppose also that we have a set of *N *proteins {*P_1_, P_2_, ... P_j_, ... P_N_*} with *q *protein domains descriptors identified in each of them respectively.

Each of the *M *drugs can therefore be represented as *D_i _*= (*sub_i1_, sub_i2_,..., sub_ix_,..., sub_ip_*), where *sub_ix _*is the *x*th substructure of the *i*th drug where *i*∈{*1, 2,..., M*} and *x*∈*{1, 2, ..., p*} and *sub_ix _*= 1 when the *i*th substructure exists in the drug, otherwise *sub_ix _*= 0. Similarly, each protein can be represented as *P_j _*= (*dom_j1_*, *dom_j2_*,...,*dom_jy_*,...,*dom_jq _*), where *dom_jy _*is the *y*th protein domain of the *j*th protein, *j*∈{*1, 2, ..., N*}, *y*∈{*1,2,..., q*} and *dom_jy _*= 1 when the *y*th protein domain *dom_jy _*exists in the protein, otherwise *dom_jy _*= 0. The existence of one of more interaction relationships between the given drugs and proteins are represented by a matrix ***I ***= (*α_1_, α_2_,..., α_M_*)^T^, where *α_i _*= (*α_i1_, α_i2_,...α_lk_,...α_iN_*), *l*∈{*1, 2, ..., M}*, *k*∈{*1, 2,..., N}*. *α_lk _*= 1 when there is an interaction between the *l*th drug and *k*th protein.

### Discovering interesting association patterns

To determine whether or not the *i*th substructure of a drug has a sufficiently strong interdependency relationship with the *j*th protein domain of proteins, we construct a contingency table (Table 1) of *P *rows and *Q *columns.

**Table 1:** Observed drug substructures and protein domains occurrence

	*dom_1_*	*dom_2_*	*...*	*dom_j_*	*...*	*dom_Q_*
***sub_1_***	*occ_11_*	*occ_12_*	*...*	*occ_1j_*		*occ_1Q_*
***sub_2_***	*occ_21_*	*occ_22_*	*...*	*occ_2j_*		*occ_2Q_*
***...***						
***sub_i_***	*occ_i1_*	*occ_i2_*	*...*	*occ_ij_*		*occ_iQ_*
***...***						
***sub_P_***	*occ_p1_*	*occ_p2_*	*...*	*occ_pj_*		*occ_pQ_*

Here in this table, *occ_ij _*denotes the number of occurrences of the case when *sub_i _*and *dom_j _*both takes on the value 1 in ***I***. Let $exp_{ij}=\frac{occ_{i+}occ_{+j}}{T}$ be the expected number of *occ_ij_*, where $occ_{i+}=\sum_{k=1}^{Q}occ_{ik}$ and $occ_{+j}=\sum_{k=1}^{P}occ_{kj}$ and $T=\sum_{l,k}occ_{lk}$. An interdependency relationship between them is considered to exist if *occ_ij _*is significantly different from *exp_ij_*. To decide if this is the case, the approach taken in [14] is used to calculate an *adjusted residual *test statistic:

(1) $$ad_{ij}=\frac{z_{ij}}{\sqrt{\left(1-\frac{occ_{i+}}{T}\right)\left(1-\frac{occ_{+j}}{T}\right)}}$$

where

(2) $${\text{z}}_{\text{ij}}=\frac{\text{oc}{\text{c}}_{\text{ij}}-\text{ex}{\text{p}}_{\text{ij}}}{\sqrt{\text{ex}{\text{p}}_{\text{ij}}}}$$

and $\left(1-\frac{occ_{i+}}{T}\right)\left(1-\frac{occ_{+j}}{T}\right)$ is the maximal likelihood of *z_ij _*defined in [15].

*ad_ij _*has an approximate normal distribution with a mean of approximately zero and a variance of approximately one. Therefore, if its absolute value exceeds 1.96, it would be considered significant at *α = 0.05 *by conventional criteria. Based on (1), we can determine if a drug substructure *sub_i _*has an interdependency relationship with the protein domain *dom_j_*, at the 95% confidence level.

It should be noted that the value of *ad_ij _*can be positive and negative. When *ad_ij _*is positive, *sub_i _*and *dom_j _*is interdepdent on each other and when *ad_ij _*is negative, they are not.

### Determining the weight of evidence for the discovered patterns

Since the existing of drug substructure in a drug is important for determining the interaction between protein domains, it is necessary to ensure that they are utilized in the prediction of an interaction relationship between a drug and a protein. The interdependency relationships discovered by (2) determines only the interdependency between drug substructures and protein domains, but it does not measure how strong the interdependency is. For this reason, we introduce the *weight of evidence *measure for the patterns discovered above.

Suppose that *dom_j _*= 1 is found to be interdependent with *sub_i _*= 1. Then the weight of evidence provided by *sub_i _*= 1 in favor of *dom_j _*= 1 opposed to *dom_i _*= 0 can be defined as [16]:

(3) $$\begin{array}{c} WoE\left(\frac{dom_{j}=1}{dom_{j}=0}:sub_{i}=1\right)\\ \;\;\;\;\;=I\left(dom_{j}=1:sub_{i}=1\right)-I\left(dom_{j}=0:sub_{i}=1\right) \end{array}$$

where

(4) $$I\left(dom_{j}=1\;:sub_{i}=1\right)\;=\;log\;\frac{Pr\text{(}dom_{j}=1\text{|}sub_{i}=1)}{\text{Pr}(dom_{j}=1)}$$

(5) $$I\left(dom_{j}=0\;:sub_{i}=1\right)\;=\;log\;\frac{Pr\text{(}dom_{j}=0\text{|}sub_{i}=1)}{\text{Pr}(dom_{j}=0)}$$

*WoE *can be used to be a positive or negative measurement for supporting or refuting the existence of an interaction relationship between a drug containing *sub_i _*and a protein containing *dom_j _*to have an interaction relationship. Hence, for a drug to be predicted to interact with a target protein, it should have sufficient support from its substructures in the sense that they should have a large enough degree of interdependency with the protein domains of the target protein.

### Evaluation of *D-PIA*

One way to evaluate the effectiveness of *D-PIA *is to see if it can correctly predict drug-protein interactions that it has no previous knowledge of. Here we propose to evaluate *D-PIA *by testing it to see if it can predict known drug-target interactions correctly.

Given a pair of drug *D_i _*and protein *P_j_*, the potential interaction between them can be estimated by determining if there is any significant interdependency between the substructures in *D_i _*and the protein domains in *P_j_*. To do so, let us denote the set of substructures in *D_i _*as *DS_i _*= {*s_1 _*, *s_2 _*, ..., *s_a_*} and the set of domains in *P_j _*as *PD_j _*= {*d_1 _*, *d_2 _*, ..., *d_b_*}, where *a *is the total number of substructures in *D_i_*, and *b *is the total number of protein domains in *P_j_*. For ∀*s*' ∈ *DS_i _*∀ *d*' ∈ *PD_i_*, we consider the interaction between *s*' and *d*' as significant when (6) below is satisfied.

(6) $$|ad_{s^{\prime }d^{\prime }}|>1.96$$

For a pair of *D_i _*and *P_j_*, there are *a *× *b *possible significant interdependency relationship of substructures and protein domains in total. The potential interaction between *D_i _*and *P_j _*can be estimated based on the interacting substructures between them. If there is only 1 significant interdependency between the substructures of a drug and protein out of the total *a *× *b *such possible relationships, we may consider that the potential interaction between *D_i _*and *P_j _*as very weak. On the other hand, if more than half of the associations are significant, we may consider that the potential interaction between *D_i _*and *P_j _*as high. Therefore we could assert that there is potential interaction between *D_i _*and *P_j _*as (7).

(7) $$w\left(D_{i},P_{j}\right)=\frac{\Sigma_{i,j}val\left(ad_{s_{j}^{\prime }d_{j}^{\prime }}\right)}{a\times b}$$

where *val(x) = 1 *if |*x*| > 1.96, otherwise *val(x) = *0.

The interaction between the drug, *D_i_*, and the protein, *P_j_*, will be more significant if the value of *w*(*D_i_*, *P_j_*) is higher than some user-supplied threshold, denoted as *R*, i.e if *w*(*D_i_*, *P_j_*) >*R*, and if, at the same time, the *WoE*(*D_i_*, *P_j_*) is also high, then it means that the interaction between *D_i _*and *P_j _*is not only just strong, but the strong interaction relationship is also supported with strong evidence.

## Results

**Figure 1:**
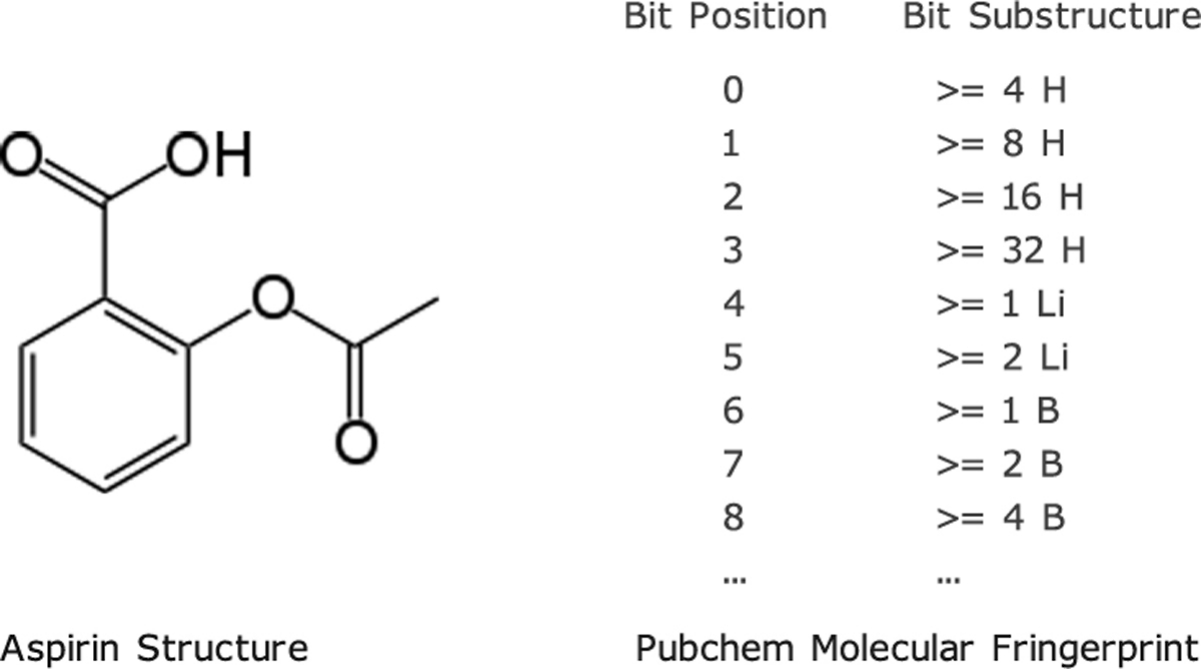
**Drug Structure and PubChem Molecular Fingerprint**.

To evaluate the effectiveness of *D-PIA*, we used the dataset from [2] which contains information about 1862 drugs. Each drug in the dataset is represented by a fingerprint with 881 substructures as defined in the PubChem database [17], i.e., each drug can be encoded as a binary vector whose elements encode for the presence or absence of a chemical substructure using 1 and 0, respectively. An example of the fingerprint of such a substructure is given in Figure 1.

**Figure 2:**
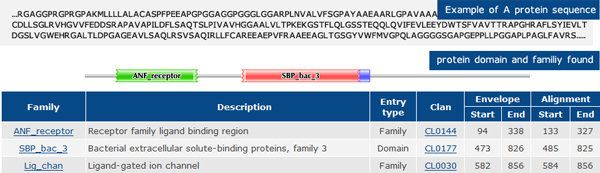
**An example of protein sequence and its protein domains **[20].

Other than the drugs, the dataset also contains information about 1554 proteins in total. According to the UniProt [18] and Pfam database [19], each of them contains a total of 876 protein domains and thus, each protein can be encoded as a binary vector whose elements encode for the presence or absence of a protein domain using 1 and 0 respectively. An example of the protein sequence and its protein domains is given in Figure 2.

Given the drugs and proteins as described above, *D-PIA *determines the *adjusted residuals *for the drug substructures and protein domains based on Equation (2) above. In Table 2, we list some of the adjusted residuals that *D-PIA *computes to determine if there is significant interdependency relationship between a drug substructure and a protein domain. As shown in the table, for example, the drug substructures of SUB840, SUB841, SUB861 are interdependent with the protein domains of PF00104 and PF00105.

**Table 2:** Some examples of adjusted residuals

	PF00102	PF00104	PF00105	PF00106	PF00107
**SUB840**	-1.447	**19.180**	**18.928**	-0.997	-1.282
**SUB841**	-1.441	**18.609**	**18.354**	-0.361	-1.276
**SUB843**	-0.381	-0.793	-0.790	-0.427	-0.338
**SUB846**	-0.337	-0.702	-0.699	-0.378	-0.299
**SUB848**	-0.159	-0.331	-0.330	-0.178	-0.141
**SUB861**	-1.673	**20.748**	**20.551**	1.901	-1.481

To evaluate the effectiveness of *D-PIA*, we therefore try to determine if there is a strong enough drug-protein interaction between the drugs *D_i _*and the protein *P_j _*in our dataset based on the *adjusted residuals *obtained between the substructures of the drugs and the protein domains of the proteins as illustrated in Table 2. We set *R *to 10% in our experiments and found *D-PIA *to be able to predict the existence of Drug-Protein interaction at an accuracy of 85.4%. A 5-fold cross-validation approach is used to evaluate the ability of *D-PIA *to determine if a drug interacts with a protein and this approach is described as follows:

1) We split the drug-protein interactions dataset into five subsets of equal size and take each subset in turn as a test set.

2) We perform *D-PIA *on the remaining 4 sets.

3) Based on the significant interdependency relationships determined between drug substructures and protein domains, *D-PIA *attempts to predict the existence of interactions between drug and protein in the testing data and the accuracy over the five folds are computed.

**Figure 3:**
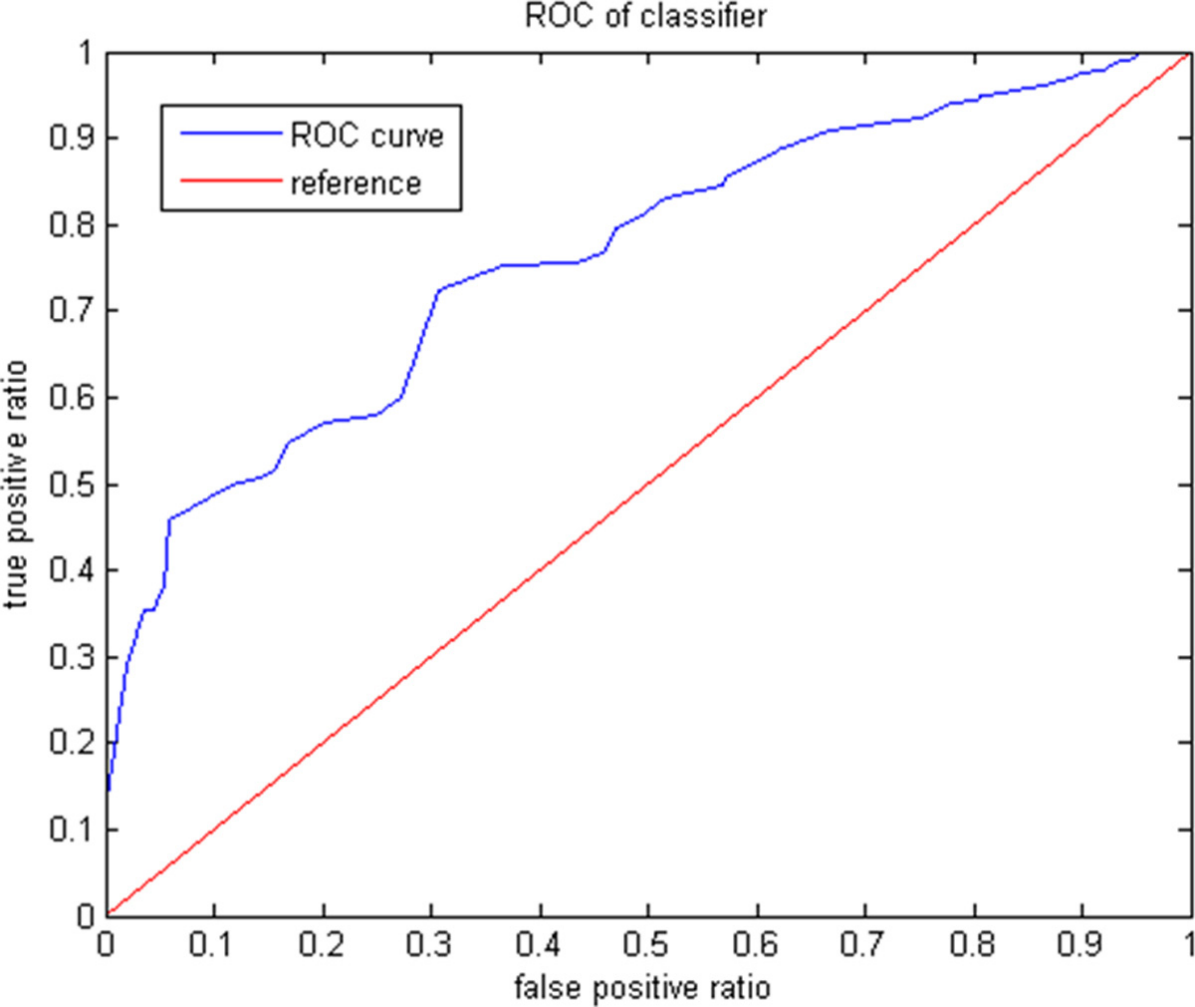
**ROC curve for the experiments**.

A ROC (receiver operating characteristic) curve [21] based on the experimental results can be obtained as shown in Figure 3.

While *w*(*D_i_*, *P_j_*) represents the existence of a significant interdependency relationship between a drug substructure *sub_m _*and a protein domain *dom_n_*, it does not tell us how strong the interdependency relationship is. To find out, we compute, as discussed above, the *WoE*(*sub_m_*, *dom_n_*) measure for the interaction between *sub_m _*and *dom_n_*. We summarize the result of the interaction between the drug substructures *sub_m _*and protein domain *dom_n _*and we present some of the results in Table 3.

**Table 3:** High value of adjust residual and *WoE *for drug protein substructures interactions

**Drug sub ref**.	Drug substructures and description	Protein dom. ref	Adjust residual	Weight of evidence
...	...	...	...	...

SUB17	>=8N	PF00156	4.303	2.034
		PF00206	3.137	2.359
		PF00583	3.279	2.447
		PF00858	5.047	2.12

...	...	...	...	...

SUB33	>=1 S	PF00017	5.553	4.118
		PF00018	3.073	3.506
		PF00069	2.363	2.193
		PF00169	23.046	7.078

...	...	...	...	...

SUB190	>=2 unsaturated non-aromatic nitrogen-containing ring size 6	PF00019	8.96	6.376
		PF00020	5.22	4.874
		PF00071	7.161	4.254
		PF00432	9.429	5.545

...	...	...	...	...

SUB235	>=2 saturated or aromatic carbon-only ring size 8	PF00020	12.08	7.214
		PF00091	5.49	4.089
		PF00531	6.814	5.599
		PF02180	14.706	6.787

...	...	...	...	...

SUB334	C(~C) (~C) (~C) (~C)	PF00133	3.605	1.992
		PF02145	2.081	2.669
		PF02188	2.081	2.669
		PF08264	3.301	1.998

...	...	...	...	...

SUB428	C(#C)(-H)	PF00039	5.319	4.926
		PF00048	1.582	2.094
		PF00104	11.315	2.849
		PF00105	11.37	2.859

...	...	...	...	...

SUB695	O=C-C-C-C-C-N	PF00120	8.354	4.686
		PF04960	9.591	4.671

...	...	...	...	...

SUB707	O=C-C-C-C-C(N)-C	PF00040	2.742	3.236
		PF00091	29.218	5.237
		PF00183	4.55	3.619
		PF00340	4.708	4.597
		PF00341	2.886	3.357

...	...	...	...	...

			29.218	7.214
			1.582	1.992

## Discussion

The ROC in Figure 3 is a chart of *true-positive *vs *false-positive *for the prediction results of the experiments. The true-positive is concerned with the rate of correctly predicted drug-protein interactions whereas the false-positives is concerned with the rate of incorrectly predicted drug-protein interactions.

We can see from the chart that *D-PIA *can be very accurate in predicting drug-protein interactions most of the ROC curve is much above the reference line (random prediction). The AUC (area under the ROC curve) score (which is 1 for perfect accuracy and 0.5 for random prediction) score for *D-PIA *is 0.7497 which shows that that it is much better than prediction at random.

These results show that *D-PIA *can be used to predict how likely a drug candidate may interact with a particular protein. Based on the *WoE *computed as shown in Table 3, we also know that candidate drugs that have the substructures SUB695 are significantly interdependent with the protein domains PF04960, etc., and we believe that the interdependency relationships and the WoE measures between them such as shown in Table 3 could be very useful for the drug discovery, pharmacological analysis, ligand specificity, etc.

## Conclusions

One common approach to drug discovery is to tackle the protein-ligand docking problem. To effectively do so, there is a need for information related to the 3D structures to be known. As such information is difficult and expensive to obtain, *D-PIA *is proposed here to discover patterns in known drug-protein interaction to predict those that are unknown so that the protein-ligand docking problem can be more easily tackled without having to rely on any 3D information. *D-PIA *makes use of fingerprints of the known drug substructures and protein domains to infer the existence of interactions between corresponding drugs and proteins. Experimental results show that the *D-PIA *can work effectively and can infer drug-protein interaction with high accuracy and can be a promising tool for computer aided drug discovery.

## Competing interests

The authors declare that they have no competing interests.

## Authors' contributions

Weimin Luo carried out this study and drafted the manuscript. Keith CC Chan conceived this study and participated in its design and helped to draft the manuscript. All the authors read and approved the final manuscript.
